# Bis[(5-phenyl-1,3,4-thia­diazol-2-yl)sulfan­yl]methane

**DOI:** 10.1107/S1600536810044442

**Published:** 2010-11-06

**Authors:** He-wen Wang, Yan Gao, Wei Wang

**Affiliations:** a College of Chemistry and Applied Chemistry, Huanggang Normal University, Huanggang 438000, People’s Republic of China; b School of Chemical Engineering, University of Science and Technology LiaoNing, Anshan 114051, People’s Republic of China; c School of Perfume and Aroma Technology, Shanghai Institute of Technology, Shanghai 200235, People’s Republic of China

## Abstract

The asymmetric unit of the title compound, C_17_H_12_N_4_S_4_, contains one half-mol­ecule situated on a twofold rotational axis. In the mol­ecule, the thia­diazole and attached phenyl rings are twisted by 5.8 (3)°.

## Related literature

For biological activity of 1,3,4-thia­diazole derivatives, see: Nakagawa *et al.* (1996 ▶); Wang *et al.* (1999 ▶); Carvalho *et al.* (2004 ▶); Riente *et al.* (2009 ▶); Poorrajab *et al.* (2009 ▶).
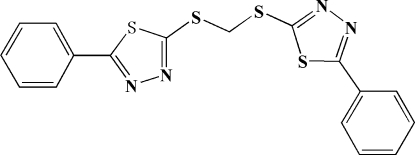


## Experimental

### 

#### Crystal data

C_17_H_12_N_4_S_4_
*M* *_r_* = 400.55
Orthorhombic, 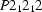

*a* = 10.805 (2) Å
*b* = 19.287 (4) Å
*c* = 4.0738 (8) Å
*V* = 848.9 (3) Å^3^
*Z* = 2
Mo *K*α radiation
μ = 0.57 mm^−1^
*T* = 113 K
0.20 × 0.18 × 0.10 mm

#### Data collection

Rigaku Saturn CCD area-detector diffractometer
Absorption correction: multi-scan (*CrystalClear*; Rigaku/MSC, 2005 ▶) *T*_min_ = 0.895, *T*_max_ = 0.945
6754 measured reflections
1477 independent reflections
1421 reflections with *I* > 2σ(*I*)
*R*_int_ = 0.028

#### Refinement

*R*[*F* ^2^ > 2σ(*F* ^2^)] = 0.029
*wR*(*F* ^2^) = 0.124
*S* = 1.03
1477 reflections
115 parameters
H-atom parameters constrained
Δρ_max_ = 0.54 e Å^−3^
Δρ_min_ = −0.55 e Å^−3^
Absolute structure: Flack (1983 ▶), 554 Friedel pairs
Flack parameter: 0.16 (14)

### 

Data collection: *CrystalClear* (Rigaku/MSC, 2005 ▶); cell refinement: *CrystalClear*; data reduction: *CrystalClear*; program(s) used to solve structure: *SHELXS97* (Sheldrick, 2008 ▶); program(s) used to refine structure: *SHELXL97* (Sheldrick, 2008 ▶); molecular graphics: *SHELXTL* (Sheldrick, 2008 ▶); software used to prepare material for publication: *SHELXTL*.

## Supplementary Material

Crystal structure: contains datablocks global, I. DOI: 10.1107/S1600536810044442/cv2783sup1.cif

Structure factors: contains datablocks I. DOI: 10.1107/S1600536810044442/cv2783Isup2.hkl

Additional supplementary materials:  crystallographic information; 3D view; checkCIF report

## Figures and Tables

**Fig. 1.**
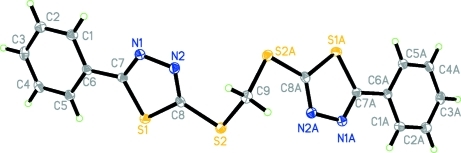
View of (I) showing the atom-labelling scheme and 35% probability displacement ellipsoids [symmetry code: (A) = -x,-y + 1,z].

## supplementary crystallographic information

### Comment

1,3,4-Thiadiazole derivatives attracted considerable attention due to their broad spectrum of chemical and pharmaceutical properties (Nakagawa *et al.*, 1996; Wang *et al.*, 1999), with particular attention being paid to the anti-trypanosomal activities of Megazol and related compounds (Carvalho *et al.*, 2004; Riente *et al.*, 2009; Poorrajab *et al.*, 2009). Herewith we report the synthesis and crystal structure of the title compound, (I), a new 1,3,4-thiadiazole derivative.

The molecular structure of (I) is shown in Fig.1. In the crystal structure, the molecule is situated on a two-fold rotational axis so asymmetric unit contains a half of the molecule. 1,3,4-Thiadiazole ring is planar with an r.m.s. deviation of 0.0048 (2)Å and maximum deviation of 0.0072 (2)Å for atom C7. The dihedral angle between the thiadiazole and attached phenyl rings is 5.8 (3)°. As a result of π-π conjugation, the C*_sp_*^2^-S bond length [S2—C8 = 1.751 (3) Å] is significantly shorter than the C*_sp_*^3^-S bond length [S2—C9 = 1.810 (2) Å].

### Experimental

A suspension of 5-diphenyl-1,3,4-thiadiazol-2-thiol (2.0 mmol) and 1,1-dibromomethane (1.0 mmol) in ethanol (10 ml) was stirred at room temperature. The reaction progress was monitored *via* TLC. The resulting precipitate was filtered off, washed with cold ethanol, dried and purified to give the target product as light yellow solid in 95% yield. Crystals of (I) suitable for single-crystal X-ray analysis were grown by slow evaporation of a solution in chloroform-ethanol (1:1).

### Refinement

All H atoms were positioned geometrically and refined as riding (C—H = 0.95–0.99 Å) and allowed to ride on their parent atoms, with *U*_iso_(H) = 1.2*U*_eq_(parent).

### Crystal data

**Table:**

C_17_H_12_N_4_S_4_	*F*(000) = 412
*M**_r_* = 400.55	*D*_x_ = 1.567 Mg m^−^^3^
Orthorhombic, *P*2_1_2_1_2	Mo *K*α radiation, λ = 0.71073 Å
Hall symbol: P 2 2ab	Cell parameters from 2856 reflections
*a* = 10.805 (2) Å	θ = 2.1–27.9°
*b* = 19.287 (4) Å	µ = 0.57 mm^−^^1^
*c* = 4.0738 (8) Å	*T* = 113 K
*V* = 848.9 (3) Å^3^	Prism, colourless
*Z* = 2	0.20 × 0.18 × 0.10 mm

### Data collection

**Table:**

Rigaku Saturn CCD area-detector diffractometer	1477 independent reflections
Radiation source: rotating anode	1421 reflections with *I* > 2σ(*I*)
confocal	*R*_int_ = 0.028
Detector resolution: 7.31 pixels mm^-1^	θ_max_ = 25.0°, θ_min_ = 2.1°
φ and ω scans	*h* = −12→12
Absorption correction: multi-scan (*CrystalClear*; Rigaku/MSC, 2005)	*k* = −22→22
*T*_min_ = 0.895, *T*_max_ = 0.945	*l* = −4→4
6754 measured reflections	

### Refinement

**Table:**

Refinement on *F*^2^	Hydrogen site location: inferred from neighbouring sites
Least-squares matrix: full	H-atom parameters constrained
*R*[*F*^2^ > 2σ(*F*^2^)] = 0.029	*w* = 1/[σ^2^(*F*_o_^2^) + (0.110*P*)^2^] where *P* = (*F*_o_^2^ + 2*F*_c_^2^)/3
*wR*(*F*^2^) = 0.124	(Δ/σ)_max_ = 0.001
*S* = 1.02	Δρ_max_ = 0.54 e Å^−^^3^
1477 reflections	Δρ_min_ = −0.55 e Å^−^^3^
115 parameters	Extinction correction: *SHELXL97* (Sheldrick, 2008), Fc^*^=kFc[1+0.001xFc^2^λ^3^/sin(2θ)]^-1/4^
0 restraints	Extinction coefficient: 0.049 (10)
Primary atom site location: structure-invariant direct methods	Absolute structure: Flack (1983), 554 Friedel pairs
Secondary atom site location: difference Fourier map	Flack parameter: 0.16 (14)

### Special details

**Table:**

Geometry. All e.s.d.'s (except the e.s.d. in the dihedral angle between two l.s. planes) are estimated using the full covariance matrix. The cell e.s.d.'s are taken into account individually in the estimation of e.s.d.'s in distances, angles and torsion angles; correlations between e.s.d.'s in cell parameters are only used when they are defined by crystal symmetry. An approximate (isotropic) treatment of cell e.s.d.'s is used for estimating e.s.d.'s involving l.s. planes.
Refinement. Refinement of *F*^2^ against ALL reflections. The weighted *R*-factor *wR* and goodness of fit *S* are based on *F*^2^, conventional *R*-factors *R* are based on *F*, with *F* set to zero for negative *F*^2^. The threshold expression of *F*^2^ > σ(*F*^2^) is used only for calculating *R*-factors(gt) *etc*. and is not relevant to the choice of reflections for refinement. *R*-factors based on *F*^2^ are statistically about twice as large as those based on *F*, and *R*- factors based on ALL data will be even larger.

### Fractional atomic coordinates and isotropic or equivalent isotropic displacement parameters (Å^2^)

**Table:**

	*x*	*y*	*z*	*U*_iso_*/*U*_eq_	Occ. (<1)
S1	0.19317 (6)	0.33910 (3)	0.3294 (2)	0.0215 (3)	
S2	0.13644 (6)	0.47579 (4)	0.6577 (2)	0.0216 (3)	
N1	−0.0251 (2)	0.29797 (13)	0.4770 (7)	0.0223 (6)	
N2	−0.0161 (2)	0.36481 (13)	0.6002 (7)	0.0231 (6)	
C1	−0.0141 (3)	0.16581 (16)	0.1495 (9)	0.0260 (7)	
H1	−0.0904	0.1795	0.2453	0.031*	
C2	−0.0048 (3)	0.10272 (16)	−0.0079 (9)	0.0289 (8)	
H2	−0.0748	0.0731	−0.0206	0.035*	
C3	0.1073 (3)	0.08236 (15)	−0.1485 (9)	0.0279 (7)	
H3	0.1136	0.0389	−0.2568	0.033*	
C4	0.2085 (3)	0.12546 (15)	−0.1299 (9)	0.0262 (7)	
H4	0.2847	0.1117	−0.2264	0.031*	
C5	0.1999 (3)	0.18852 (16)	0.0284 (8)	0.0234 (7)	
H5	0.2704	0.2178	0.0420	0.028*	
C6	0.0885 (3)	0.20960 (14)	0.1682 (8)	0.0196 (6)	
C7	0.0747 (2)	0.27771 (15)	0.3277 (7)	0.0181 (6)	
C8	0.0919 (3)	0.39217 (14)	0.5395 (8)	0.0192 (7)	
C9	0.0000	0.5000	0.8888 (11)	0.0229 (10)	
H9A	0.0223	0.5394	1.0330	0.027*	0.50
H9B	−0.0223	0.4606	1.0330	0.027*	0.50

### Atomic displacement parameters (Å^2^)

**Table:**

	*U*^11^	*U*^22^	*U*^33^	*U*^12^	*U*^13^	*U*^23^
S1	0.0163 (4)	0.0229 (4)	0.0252 (5)	−0.0010 (3)	0.0012 (4)	−0.0011 (3)
S2	0.0221 (4)	0.0204 (4)	0.0223 (5)	−0.0011 (3)	−0.0018 (4)	0.0005 (3)
N1	0.0199 (12)	0.0197 (12)	0.0273 (14)	0.0005 (9)	−0.0006 (12)	0.0018 (12)
N2	0.0229 (12)	0.0200 (12)	0.0263 (15)	0.0006 (10)	0.0035 (11)	−0.0007 (12)
C1	0.0198 (14)	0.0279 (15)	0.0303 (18)	0.0002 (11)	0.0039 (16)	0.0029 (17)
C2	0.0258 (14)	0.0254 (15)	0.036 (2)	−0.0019 (12)	−0.0060 (17)	−0.0012 (16)
C3	0.0374 (17)	0.0206 (13)	0.0257 (17)	0.0052 (13)	0.0029 (17)	−0.0019 (15)
C4	0.0244 (14)	0.0254 (14)	0.0289 (18)	0.0079 (12)	0.0013 (15)	0.0031 (16)
C5	0.0208 (14)	0.0232 (14)	0.0260 (17)	0.0012 (12)	−0.0002 (15)	0.0041 (14)
C6	0.0189 (14)	0.0209 (14)	0.0191 (15)	0.0028 (11)	−0.0046 (13)	0.0043 (14)
C7	0.0162 (13)	0.0216 (13)	0.0166 (14)	0.0000 (11)	−0.0015 (13)	0.0033 (13)
C8	0.0206 (14)	0.0198 (12)	0.0170 (15)	0.0038 (11)	−0.0010 (13)	−0.0001 (12)
C9	0.029 (2)	0.0235 (19)	0.016 (2)	−0.0003 (17)	0.000	0.000

### Geometric parameters (Å, °)

**Table:**

S1—C8	1.726 (3)	C2—H2	0.9500
S1—C7	1.744 (3)	C3—C4	1.376 (5)
S2—C8	1.751 (3)	C3—H3	0.9500
S2—C9	1.810 (2)	C4—C5	1.380 (5)
N1—C7	1.298 (4)	C4—H4	0.9500
N1—N2	1.387 (4)	C5—C6	1.391 (4)
N2—C8	1.304 (4)	C5—H5	0.9500
C1—C2	1.379 (5)	C6—C7	1.473 (4)
C1—C6	1.396 (4)	C9—S2^i^	1.810 (2)
C1—H1	0.9500	C9—H9A	0.9900
C2—C3	1.396 (4)	C9—H9B	0.9900
			
C8—S1—C7	86.51 (14)	C4—C5—H5	119.8
C8—S2—C9	99.02 (10)	C6—C5—H5	119.8
C7—N1—N2	113.0 (2)	C5—C6—C1	119.2 (3)
C8—N2—N1	111.8 (2)	C5—C6—C7	121.9 (3)
C2—C1—C6	120.1 (3)	C1—C6—C7	118.9 (3)
C2—C1—H1	119.9	N1—C7—C6	124.0 (3)
C6—C1—H1	119.9	N1—C7—S1	113.8 (2)
C1—C2—C3	120.1 (3)	C6—C7—S1	122.2 (2)
C1—C2—H2	119.9	N2—C8—S1	114.9 (2)
C3—C2—H2	119.9	N2—C8—S2	124.5 (2)
C4—C3—C2	119.8 (3)	S1—C8—S2	120.57 (17)
C4—C3—H3	120.1	S2—C9—S2^i^	117.3 (2)
C2—C3—H3	120.1	S2—C9—H9A	108.0
C3—C4—C5	120.3 (3)	S2^i^—C9—H9A	108.0
C3—C4—H4	119.9	S2—C9—H9B	108.0
C5—C4—H4	119.9	S2^i^—C9—H9B	108.0
C4—C5—C6	120.5 (3)	H9A—C9—H9B	107.2
			
C7—N1—N2—C8	−0.5 (4)	C1—C6—C7—N1	5.7 (5)
C6—C1—C2—C3	0.1 (5)	C5—C6—C7—S1	4.3 (4)
C1—C2—C3—C4	0.0 (5)	C1—C6—C7—S1	−173.9 (3)
C2—C3—C4—C5	0.3 (6)	C8—S1—C7—N1	−1.1 (2)
C3—C4—C5—C6	−0.6 (5)	C8—S1—C7—C6	178.6 (3)
C4—C5—C6—C1	0.7 (5)	N1—N2—C8—S1	−0.4 (3)
C4—C5—C6—C7	−177.6 (3)	N1—N2—C8—S2	179.9 (2)
C2—C1—C6—C5	−0.4 (5)	C7—S1—C8—N2	0.8 (3)
C2—C1—C6—C7	177.9 (3)	C7—S1—C8—S2	−179.5 (2)
N2—N1—C7—C6	−178.6 (2)	C9—S2—C8—N2	4.3 (3)
N2—N1—C7—S1	1.1 (3)	C9—S2—C8—S1	−175.34 (19)
C5—C6—C7—N1	−176.0 (3)	C8—S2—C9—S2^i^	−76.74 (11)

Symmetry codes: (i) −*x*, −*y*+1, *z*.
